# Unveiling the hidden cardiovascular risk of sipuleucel-T: a pharmacovigilance analysis using the FDA Adverse Event Reporting System, 2010–2025

**DOI:** 10.3389/fimmu.2025.1716090

**Published:** 2026-01-20

**Authors:** Wensheng Liu, Xue Song, Linlin Wang, Jiyong Liu, Qiong Du

**Affiliations:** 1 Department of Pharmacy, Fudan University Shanghai Cancer Center, Shanghai, China; 2 Department of Oncology, Shanghai Medical College, Fudan University, Shanghai, China; 3 Department of Radiation Oncology, Fudan University Shanghai Cancer Center, Shanghai, China; 4 Department of Pharmacy, Fudan University Shanghai Cancer Center Xiamen Hospital, Xiamen, China

**Keywords:** castration-resistant, disproportionality analysis, FAERS, pharmacovigilance, prostate cancer, Provenge, sipuleucel-T

## Abstract

**Background:**

Sipuleucel-T, the first therapeutic cancer vaccine approved by the U.S. Food and Drug Administration (FDA), represents a crucial treatment option for patients with metastatic castration-resistant prostate cancer (mCRPC). However, the characteristics of cardiovascular adverse events (CVAEs) associated with sipuleucel-T remain poorly understood.

**Methods:**

This retrospective, pharmacovigilance analysis used case safety reports from the FDA Adverse Event Reporting System (FAERS) spanning April 2010 to March 2025. Reporting odds ratio (ROR) and information component (IC) were applied to identify and evaluate potential CVAEs associated with sipuleucel-T. Kaplan–Meier method and Weibull distribution were used to analyze reported time-to-onset patterns of sipuleucel-T-related CVAEs. Multivariate logistic regression was employed to explore risk factors for CVAEs following sipuleucel-T treatment.

**Results:**

Among 4,797 sipuleucel-T-related reports, 743 (15.49%) documented CVAEs, of which 427 (57.5%) were classified as serious. Positive CVAE signals associated with sipuleucel-T included hypertension, venous thromboembolic events, arterial thromboembolic events, cardiac failure, cardiac arrhythmias, and myocardial infarction. The median time to onset of sipuleucel-T-related CVAEs was 14 days, with 80.11% occurring within 1 month. Moreover, the median time to onset of fatal CVAEs was significantly later than that of non-fatal CVAEs (22 days *vs.* 14 days; *p* = 0.0076). Additionally, age ≥ 75 years, body weight ≥ 75 kg, and concomitant use of ≥5 medications were identified as independent risk factors for sipuleucel-T-related CVAEs (*p* < 0.001).

**Conclusions:**

This study characterizes the clinical spectrum, time-to-onset patterns, and risk factors of sipuleucel-T-associated CVAEs, providing essential pharmacovigilance data for managing patients with mCRPC.

## 1 Introduction

In 2024, approximately 299,010 new cases of metastatic prostate cancer were recorded, accounting for 29% of all new cancer cases, with 35,250 related deaths. This contributes to a substantial global disease burden, making it the second leading cause of cancer-related deaths among men (1). In recent years, the therapeutic landscape for metastatic castration-resistant prostate cancer (mCRPC) has evolved unprecedentedly with the introduction of chemotherapeutics, androgen receptor pathway inhibitors, poly(ADP-ribose) polymerase inhibitors, radiopharmaceuticals (e.g., radium-223, ^177^Lu-PSMA-617), and immunotherapeutics (2–5). However, treatment options for patients with mCRPC remain limited, highlighting an urgent need for therapeutic advances.

Sipuleucel-T (Provenge®), the first U.S. Food and Drug Administration (FDA)-approved therapeutic cancer vaccine, represents a paradigm shift in the management of asymptomatic or minimally symptomatic mCRPC management (6, 7). By activating autologous immune cells that target prostate-specific antigen, sipuleucel-T has been shown to prolonged median overall survival (OS) by 4.1 months compared with placebo, establishing it as a promising therapeutic option, particularly for patients unable to tolerate traditional therapies or at high risk of treatment-related complications (6, 8). The most common adverse events (AEs; ≥15%) associated with sipuleucel-T include arthralgia, chills, back pain, fever, nausea, fatigue, and headache (6). Notably, in the pivotal trials, a discrepancy in cerebrovascular event incidence was observed between the sipuleucel-T and control groups (3.5% *vs.* 2.6%, respectively), though this difference did not reach statistical significance (6, 7, 9, 10). Moreover, cases of inflammatory cardiomyopathy has also been reported in association with sipuleucel-T treatment in postmarketing clinical practice (11). Taken together, these findings underscore the critical need for large-sample, real-world data to accurately characterize the risk of cardiovascular adverse events (CVAEs) associated with sipuleucel-T therapy.

Pharmacovigilance databases serve as a crucial supplementary resource for investigating potential AEs associated with marketed drugs. The U.S. FDA Adverse Event Reporting System (FAERS) is a globally recognized, authoritative, and open-access database for spontaneous reporting of adverse events from post-marketing routine clinical care (12). Given that sipuleucel-T is currently approved primarily in the U.S. market (6, 7), which allows FAERS to ensure sufficient sample size and standardized data to support the risk analysis of its related CVAEs.

In light of the above considerations, the present study aims to characterize the clinical profile, temporal onset pattern, risk factors, and management strategies of sipuleucel-T-related CVAEs leveraging the FAERS database. By identifying potential CVAE signals that may have been underreported in clinical trials, this study provides a pharmacovigilance-informed reference for therapeutic decision-making, with the goal of optimizing management strategies for patients with mCRPC.

## 2 Materials and methods

### 2.1 Data sources and pre-processing

A retrospective real-world disproportionality analysis was conducted to assess the CVAEs associated with sipuleucel-T therapy, utilizing individual case safety reports extracted from the FAERS (https://fis.fda.gov/extensions/FPD-QDE-FAERS/FPD-QDE-FAERS.html) for the period from April 1, 2010 (April 29, 2010), through March 31, 2025. These reports cover patient demographics (DEMO), indications (INDI), medication (DRUG), AEs (REAC), outcomes (OUTC), therapy start and end dates for the reported drugs (THER), and report sources (RPSR). Duplicate reports were removed following FDA-recommended methods: (1) For reports with identical CASEIDs, the one with the largest FDA_DT (report submission date) was retained; (2) For reports with identical CASEIDs and FDA_DT values, the one with the largest PRIMARYID (unique report identifier) was retained. Additionally, cases from the “Deleted Cases” file were excluded to ensure data accuracy and integrity. Simultaneously, reports in which the first medication administration date occurred after the date of the adverse event were identified as anomalous and excluded from subsequent analyses (12).

Given that patient records in FAERS are fully anonymized and de-identified, this safety evaluation was exempt from institutional review board approval as well as from informed consent. To guarantee methodological transparency and reproducibility, this study strictly adhered to the READUS-PV guideline (13, 14). Moreover, this article was prepared in compliance with the TITAN 2025 guidelines (15).

### 2.2 Identification of CVAEs reports with sipuleucel-T therapy

Only reports in which sipuleucel-T was listed as the “primary suspect” drug were included for exposure assessment. The target drug was identified by matching its generic name (sipuleucel-T) and brand name (Provenge^®^) to the “prod_ai” and “drugname” fields in the DRUG table. To systematically summarize and analyze specific CVAEs related to sipuleucel-T, all CVAEs were standardized and categorized using Medical Dictionary for Regulatory Activities (MedDRA, version 27.0) terminology. CVAEs of interest were identified using Standardized MedDRA Queries (SMQs). Cases were defined as reports containing at least one of the following CVAEs of interest: cardiac arrhythmias, cardiac failure, cardiomyopathy, hypertension, myocardial infarction, noninfectious myocarditis/pericarditis, other ischemic heart disease, pulmonary hypertension, arterial thrombotic/embolic events (ATE), unspecified or mixed arterial/venous thrombotic/embolic events (other TEs), and venous thrombotic/embolic events (VTE). Additionally, searches used narrow SMQ terms to enhance specificity and increase the positive predictive value of case identification (specific CVAE-related preferred terms (PTs) are listed in online **Supplementary Table S1**) (12).

### 2.3 Sensitivity analysis

To eliminate the influence of potential confounding factors on the results of the study and test the robustness of disproportionate signals, a series of sensitivity analyses were performed. First, when adverse events under investigation are associated with one or more drugs other than the target drug, bias from drug-drug competition may arise. Therefore, by reviewing drug labels, we excluded cases of CVAEs reported concurrently with other medications, thereby reducing competitive bias. Second, to avoid exposure bias, we only included reports identifying sipuleucel-T as the primary suspect drug. Third, we restricted the scope of the reports to those submitted by healthcare professionals to further alleviate reporting bias.

### 2.4 Global assessment of the evidence

A potential causal association between sipuleucel-T treatment and CVAEs was assessed by evaluating the entire body of evidence, using the adapted Bradford Hill Criteria—an approach widely utilized in both epidemiological and pharmacovigilance research (16, 17). To determine the strength and plausibility of the observed associations, several key dimensions of the criteria were analyzed, including biological plausibility, strength of association, consistency, specificity, coherence, and analogy. However, due to the limited availability of rechallenge and de-challenge data in the FAERS database, the “reversibility” criterion was not evaluated in this study.

### 2.5 Statistical analysis

*Descriptive statistics*: Descriptive analyses were performed to characterize the clinical profiles of patients experiencing sipuleucel-T-related CVAEs in the FAERS database. Categorical variables for all reports (both serious and nonserious) and serious reports were presented as numbers and percentages. Data quality control included the exclusion of implausible physiological values (ages >120 years, weights >400 kg). Owing to sipuleucel-T’s indication limited to prostate cancer at the time of the study, female patients were excluded (n = 1). A report was classified as serious if the patient outcome included death, hospitalization, disability, life-threatening events, intervention to prevent permanent impairment or damage, or other serious outcomes. Fatal CVAEs were defined as cases with a patient outcome of death, all other serious outcomes were classified as nonfatal.

*Disproportionality analyses*: Two specific indices, the reporting odds ratio (ROR) and BCPNN information component (IC), were conducted to evaluate the potential CVAE risk linked to sipuleucel-T therapy, with all other drugs/events documented in the FAERS database serving as the comparator. The relevant calculation formulas and criteria of ROR and IC are provided in online **Supplementary Tables S2**. Consistent with prior disproportionality analyses, a potential safety signal was considered statistically significant if three criteria were met: the lower bound of the 95% confidence interval (CI) for the ROR (ROR_025_) exceeded 1, the lower bound of the 95% CI for the IC (IC_025_) was greater than 0, and there were at least 3 case reports of specific CVAE (18).

*Time to onset analyses:* The time to onset was defined as the interval between the CVAE onset date (EVENT_DT in the DEMO file) and the sipuleucel-T initiation date (START_DT in the THER file). After excluding reports with missing or inaccurate dates, the Kaplan-Meier method was used to plot the cumulative incidence of non-fatal and fatal CVAEs related to sipuleucel-T, with statistical significance assessed via the Wilcoxon two-sample test. A *p*-value < 0.05 was considered to indicate statistical significance. Meanwhile, the shape parameter β of the Weibull distribution was used to describe the changes in the risk of CVAEs associated with sipuleucel-T therapy. Based on the value of the shape parameter β, three distinct patterns of hazard evolution are observed: if the upper limit of the 95% CI for β is < 1, this indicates a hazard that increases initially and then decreases (early failure type). Conversely, if the 95% CI for β includes 1, the hazard remains constant over the entire exposure period (random failure type). Finally, if the lower limit of the 95% CI for β is > 1, this demonstrates a hazard that increases with time (wear-out failure type) (16, 19).

*Multivariable logistic regression*: To identify independent risk factors associated with CVAEs, a multivariable logistic regression model was fitted using the glm function in R. The outcome variable was the binary occurrence of CVAE. Predictor variables—selected based on clinical relevance—included age (categorized as <75 or ≥75 years), weight (categorized as <75 or ≥75 kg), and the number of concomitant medications (categorized as 0, 1–5, or >5). Continuous variables were categorized to enhance clinical interpretability and to address potential non-linear relationships. Results are reported as adjusted odds ratios (ORs) with 95% CIs. Potential multicollinearity was examined by calculating variance inflation factors (VIFs), and all VIF values were <5 indicating no substantial collinearity among predictors. Cases with missing data for any included variable were excluded from the regression analysis.

All data management, statistical analysis, and graphical representations in this study were executed using R software, version 4.3.3 (R Foundation for Statistical Computing, Vienna, Austria).

## 3 Results

### 3.1 Descriptive analysis

**Figure 1:**
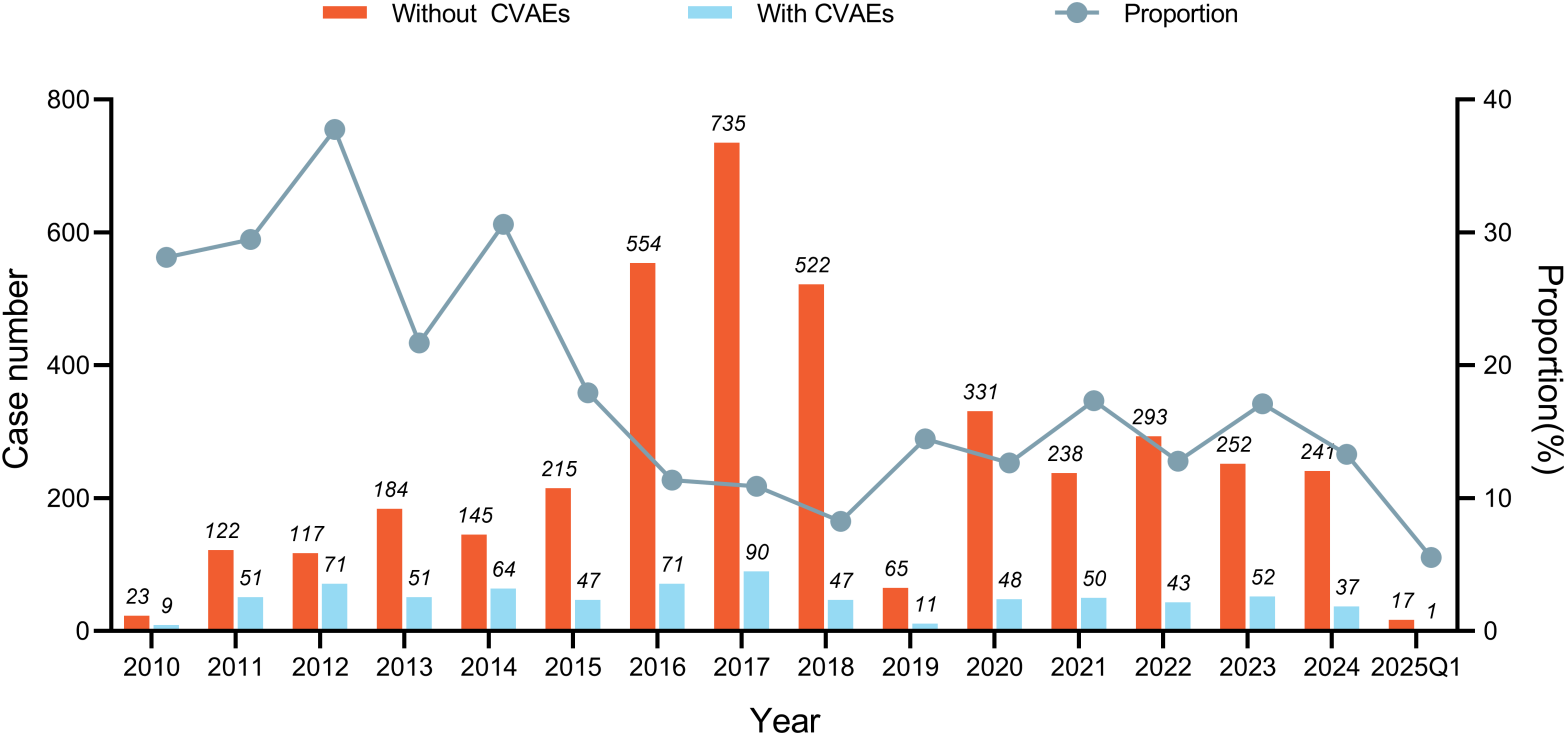
Statistics of cardiovascular reports associated with sipuleucel-T in the FAERS database from April 2010 to March 2025. The bar chart shows the annual number of reports of sipuleucel-T-related cardiovascular adverse events (CVAEs) and non-CVAEs, while the line chart depicts the annual proportion of sipuleucel-T-related CVAEs among all reported adverse events.

During the surveillance period, from April 2010 to March 2025, the annual count of reported CVAEs associated with sipuleucel-T therapy and their proportion among all adverse events varied, with an overall proportion ranging between 10% and 40% (**Figure 1**). A total of 4,797 cases were identified with sipuleucel-T as the primary suspect documented in the FAERS database, of which 743 cases (15.49%) involved reported CVAEs, and 427 were classified as serious outcomes (57.5%). The median (interquartile range [IQR]) patient age was 74 (67-80) years, and 464 patients were specified to be males. Most reports were submitted by physicians or other health care professionals (85.2%). The specific demographic and clinical details are provided in **Table 1**.

**Table 1:** Description of cardiovascular reports submitted to the US FDA Adverse Event Reporting System for sipuleucel-T therapy between April 1, 2010, and March 31, 2025.

	No. (%)	
Report	All CVAE cases	Serious CVAE cases
Total	N=743	N=427
Gender		
Male	464 (62.4%)	277 (64.9%)
Not specified	279 (37.6%)	150 (35.1%)
Weight, kg		
< 50	2 (0.3%)	2 (0.5%)
50 -100	196 (26.4%)	125 9.3%)
>100	69 (9.3%)	42 (9.8%)
Not specified	476 (64.1%)	258 (60.4%)
Age, year		
18-64	74 (10.0%)	45 (10.5%)
65-85	316 (42.5%)	192 (45.0%)
>85	38 (5.1%)	22 (5.2%)
Not specified	315 (42.4%)	168 (39.3%)
Source of report		
Physician	190 (25.6%)	124 (29.0%)
Pharmacist	15 (2.0%)	10 (2.3%)
Health Professional	185 (24.9%)	92 (21.5%)
Other health-professional	237 (31.9%)	125 (29.3%)
Registered nurse	6 (0.8%)	5 (1.2%)
Consumer	84 (11.3%)	53 (12.4%)
Not specified	26 (3.5%)	18 (4.2%)
Reported Country		
USA	722(97.2%)	414 (97.0%)
Not specified	21 (2.8%)	13 (3.0%)
Reported outcome		
Death	47 (6.3%)	47 (11.0%)
Non-fatal	380 (51.2%)	380 (89.0%)
Not specified	316 (42.5%)	0 (0%)

CVAE, cardiovascular adverse event; FDA, Food and Drug Administration; Non-fatal, hospitalization, disability, life-threatening, required intervention or other serious outcomes.

### 3.2 Signal detection for CVAEs associated with sipuleucel-T therapy

**Figure 2:**
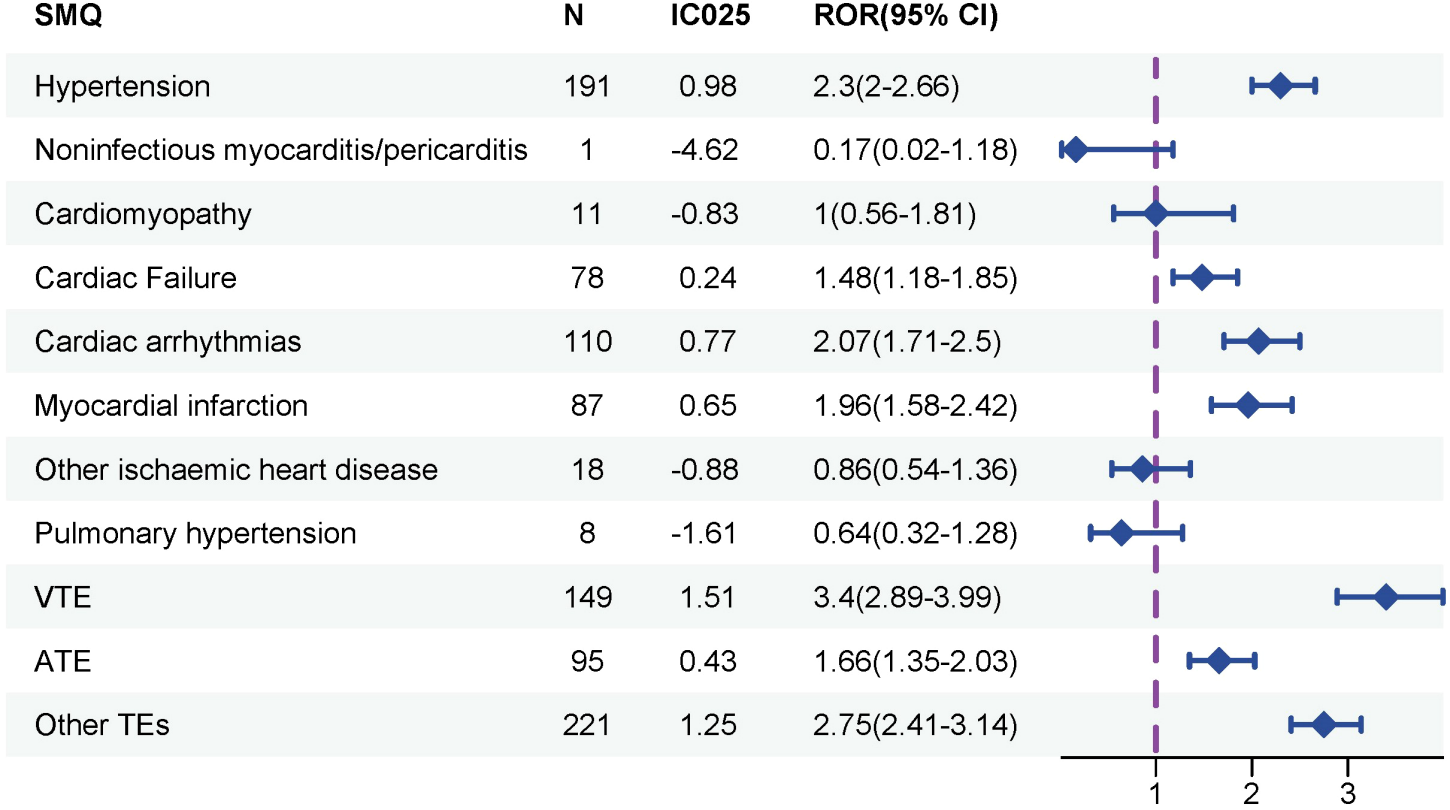
Disproportionality analysis of significant cardiovascular adverse events associated with sipuleucel-T at the Standardized MedDRA Query (SMQ) level. Signals at the SMQ level reflect the strength and statistical significance of the association between the sipuleucel-T and specific cardiovascular outcomes. A signal was considered significant if three criteria were met simultaneously: the lower limit of the reporting odds ratio (ROR) exceeded 1, the lower bound of the 95% confidence interval for the information component (IC) was greater than 0, and the number of cases was ≥3.

The disproportionality analyses of CVAEs associated with sipuleucel-T therapy are presented in **Figure 2** and **Supplementary Figure 1**. Positive signals were observed for several SMQs of CVAEs in association with sipuleucel-T therapy (**Figure 2**), including hypertension (ROR_025_ = 2; IC_025_ = 0.98), cardiac failure (ROR_025_ = 1.18; IC_025_ = 0.24), cardiac arrhythmias (ROR_025_ = 1.71; IC_025_ = 0.77), myocardial infarction (ROR_025_ = 1.58; IC_025_ = 0.65), embolic and thrombotic events, venous (VTE, ROR_025_ = 2.89; IC_025_ = 1.51), embolic and thrombotic events, arterial (ATE, ROR_025_ = 1.35; IC_025_ = 0.43) and embolic and thrombotic events, vessel type unspecified and mixed arterial and venous (Other TEs, ROR_025_ = 2.41; IC_025_ = 1.25).

Among the 743 reports, a total of 95 cardiovascular PTs associated with sipuleucel-T treatment were identified. The most frequently reported PTs included hypertension (n = 107), blood pressure increased (n = 74), deep vein thrombosis (n = 63), atrial fibrillation (n = 61), and pulmonary embolism (n = 59) (**Supplementary Table 3**). Furthermore, disproportionality analyses disclosed 27 positive signals associated with CVAEs about sipuleucel-T therapy (**Supplementary Figure 1**). In addition, the data mining analyses also detected disproportionate reporting for CVAEs not described in the U.S. package insert of sipuleucel-T (n = 17), including atrial fibrillation, heart rate irregular, atrial flutter, cardiac failure congestive, cardiac failure acute, coronary artery occlusion, troponin increased, coronary artery disease, tricuspid valve incompetence, peripheral arterial occlusive disease, jugular vein thrombosis, venous thrombosis, thrombophlebitis, device occlusion, vascular device occlusion, thrombosis in device, and monoplegia. Meanwhile, the top 3 overreporting CVAEs were vascular device occlusion (ROR_025_ = 166.61; IC_025_ = 7.29), jugular vein thrombosis (ROR_025_ = 14.1; IC_025_ = 3.84), and device occlusion (ROR_025_ = 10.98; IC_025_ = 3.44).

### 3.3 Sensitivity analysis

The list of the CVAE risks associated with the top ten concomitant medications is shown in **Supplementary Table 4**. The most frequently reported concomitant medications was leuprolide (n =106, 14.27%), aspirin (n = 92, 12.38%) and metoprolol (n =87, 11.71%).

To further evaluate the robustness of the results, we conducted a sensitivity analysis on the CVAEs signals related to sipuleucel-T. Even after adjustment for potential confounders (including competitive bias from concomitant medications and information bias from the reporter), a total of 17 clinically relevant CVAEs remained significantly associated with sipuleucel-T treatment. The disproportionate signal of sipuleucel-T-related CVAEs observed in the multiple sensitivity analyses is detailed in **Table 2**. Notable PTs included atrial fibrillation (ROR_025_ = 1.41; IC_025_ = 0.49), cardiac failure congestive (ROR_025_ = 1.84; IC_025_ = 0.88), hypertension (ROR_025_ = 2.35; IC_025_ = 1.22), acute myocardial infarction (ROR_025_ = 1.77; IC_025_ = 0.84), transient ischemic attack (ROR_025_ = 1.78; IC_025_ = 0.85), deep vein thrombosis (ROR_025_ = 2.64; IC_025_ = 1.39), pulmonary embolism (ROR_025_ = 1.62; IC_025_ = 0.69), and cerebrovascular accident (ROR_025_ = 1.03; IC_025_ = 0.04).

**Table 2:** Sensitivity analysis of cardiovascular adverse events associated with sipuleucel-T.

SMQ	PTs	Case number	ROR (95%CI)	IC025
Cardiac arrhythmias	**Atrial Fibrillation**	**25**	**2.09 (1.41, 3.09)**	**0.49**
	**Heart Rate Irregular**	**7**	**2.59 (1.23, 5.43)**	**0.35**
	Atrial Flutter	1	1.04 (0.15, 7.41)	-1.98
Cardiac Failure	**Cardiac Failure Congestive**	**27**	**2.69 (1.84, 3.92)**	**0.88**
	Cardiac Failure Acute	2	2.44 (0.61, 9.76)	-0.38
Hypertension	**Hypertension**	**76**	**2.95 (2.35, 3.7)**	**1.22**
	**Blood Pressure Increased**	**51**	**2.7 (2.05, 3.56)**	**1.02**
Myocardial infarction	**Acute Myocardial Infarction**	**11**	**3.2 (1.77, 5.79)**	**0.84**
	**Coronary Artery Occlusion**	**7**	**4.74 (2.26, 9.94)**	**1.22**
	Troponin Increased	2	2.33 (0.58, 9.32)	-0.45
Other ischemic heart disease	Coronary Artery Disease	6	1.8 (0.81, 4)	-0.25
Pulmonary hypertension	Tricuspid Valve Incompetence	1	1.22 (0.17, 8.69)	-1.75
ATE	**Transient Ischemic Attack**	**12**	**3.14 (1.78, 5.53)**	**0.85**
	Ischemic Stroke	4	1.78 (0.67, 4.74)	-0.46
	Peripheral Arterial Occlusive Disease	1	1.85 (0.26, 13.14)	-1.15
VTE	**Deep Vein Thrombosis**	**30**	**3.77 (2.64, 5.4)**	**1.39**
	**Pulmonary Embolism**	**27**	**2.36 (1.62, 3.45)**	**0.69**
	**Jugular Vein Thrombosis**	**7**	**33.56 (15.96, 70.54)**	**4.04**
	**Venous Thrombosis**	**5**	**10.55 (4.39, 25.38)**	**2.22**
	Thrombophlebitis	1	2.42 (0.34, 17.15)	-0.77
Other TEs	**Cerebrovascular Accident**	**29**	**1.49 (1.03, 2.14)**	**0.04**
	**Thrombosis**	**41**	**4.19 (3.08, 5.69)**	**1.61**
	**Device Occlusion**	**37**	**20.29 (14.68, 28.04)**	**3.86**
	**Vascular Device Occlusion**	**13**	**326.07 (186.75, 569.31)**	**7.49**
	**Thrombosis In Device**	**7**	**16.71 (7.96, 35.1)**	**3.04**
	Embolic Stroke	1	2.17 (0.3, 15.38)	-0.93
	Monoplegia	2	3.82 (0.95, 15.27)	0.26

Values in bold indicates significant signals in two algorithms. A signal was considered significant if three criteria were met simultaneously: the lower limit of the reporting odds ratio (ROR) exceeded 1, the lower bound of the 95% confidence interval for the information component (IC) was greater than 0, and the number of cases was ≥3.

### 3.4 Causal relationship global assessment

Overall, a global assessment based on the Bradford Hill Criteria supported a probable causal association between sipuleucel-T treatment and CVAEs. This conclusion is further corroborated by the robust strength of disproportionality signals, with ROR values ranging from 1.78 to 272.24, which were consistently identified using two distinct algorithms (ROR and BCPNN). Furthermore, the biological plausibility and temporal relationship underlying this association are supported by the well-established pharmacological mechanisms of sipuleucel-T (**Table 3**).

**Table 3:** Global assessment of the association between sipuleucel-T therapy and cardiovascular adverse events through adapted Bradford Hill Criteria.

Criteria	Description	Source/method
Strength of the association	Significant disproportionate signals were consistently identified for diverse CVAEs associated with sipuleucel-T therapy across different detection algorithms (ROR and IC).	Disproportionate reporting of CVAEs with sipuleucel-T in the FAERS database
Analogy	Similar cerebrovascular event and cardiac events have been observed with in previous randomized controlled trials and post-marketing case reports.	Literature and FDA labels
Biological plausibility/ empirical evidence	Sipuleucel-T exerts its therapeutic effect by harnessing the patient’s own immune system to recognize and eliminate prostate cancer cells. Sipuleucel-T could trigger an inflammatory cascade, which can further lead to T-cell recruitment, as well as the induction of myocardial injury and development of thrombosis.	Literature
Consistency	Observational studies or published case reports support the potential association of cardiovascular risks with sipuleucel-T.	Literature
Specificity	Pharmacovigilance data suggest a stronger association between sipuleucel-T and CVAEs compared to other drugs.	Disproportionality analysis
Temporal relationship	Time-to-onset analysis indicated an early failure-type pattern was identified for sipuleucel-T-related CVAEs, with the highest risk occurring during the initial treatment period. Moreover, the median time to onset of fatal CVAEs was significantly later than that of non-fatal CVAEs.	Time-to-onset analysis
Reversibility	In the context of this study, the applicability of this criterion is constrained, primarily owing to the lack of rechallenge and de-challenge data within the FAERS database.	Not applicable
Coherence	Findings align with established knowledge (e.g. randomized controlled trials and post-marketing case reports) regarding the potential association of cardiovascular risks with sipuleucel-T therapy.	Literature

CVAEs, cardiovascular adverse events; FAERS, the U.S. Food and Drug Administration Adverse Event Reporting System; ROR, reporting odds ratio; IC, information component.

### 3.5 Risk factors for sipuleucel-T therapy−associated CVAEs

In this study, a multivariable logistic regression analysis was conducted to explore the OR of sipuleucel-t therapy-related CVAEs, with adjustments for demographic and medication factors (**Table 4**). When examining demographic factors, age ≥ 75 years (OR = 1.61, 95% CI: 1.46-1.79; *p* < 0.001) and weight ≥ 75 kg (OR = 1.42, 95% CI: 1.23-1.63; *p* < 0.001) were identified as significant predictors for sipuleucel-T therapy−associated CVAEs. Regarding concomitant medications, using more than 5 concomitant drugs group was associated with an increased risk of CVAEs (OR = 1.77, 95% CI: 1.49-2.13; *p* < 0.001) compared to the reference category (0 concomitant drugs), whereas the group of 1–5 concomitant drugs showed no significant association (OR = 1.05, 95% CI: 0.87-1.29; *p* = 0.605).

**Table 4:** Multivariable logistic regression analysis of the odds ratio for sipuleucel-T therapy related cardiovascular adverse events controlling for demographic and medicine factors.

Predictor variables	Estimate	Std. Error	z value	OR(95%CI)	*p* -value
Intercept	-1.716	0.109	-15.714	–	–
Age ≥ 75 years	0.479	0.052	9.248	1.61 (1.46-1.79)	<0.001
Weight ≥ 75 kg	0.349	0.072	4.859	1.42 (1.23-1.63)	<0.001
Co-drugs: 1-5	0.052	0.101	0.517	1.05 (0.87-1.29)	0.605
Co-drugs: > 5	0.573	0.091	6.285	1.77 (1.49-2.13)	<0.001

The analysis adjusts for age (reference: < 75 years), weight (reference: < 75 kg) and number of concomitant drugs (reference: none); -, NA; OR, odds ratio; CI, confidential interval.

### 3.6 Time-to-onset analysis of sipuleucel-T therapy−associated CVAEs

**Figure 3:**
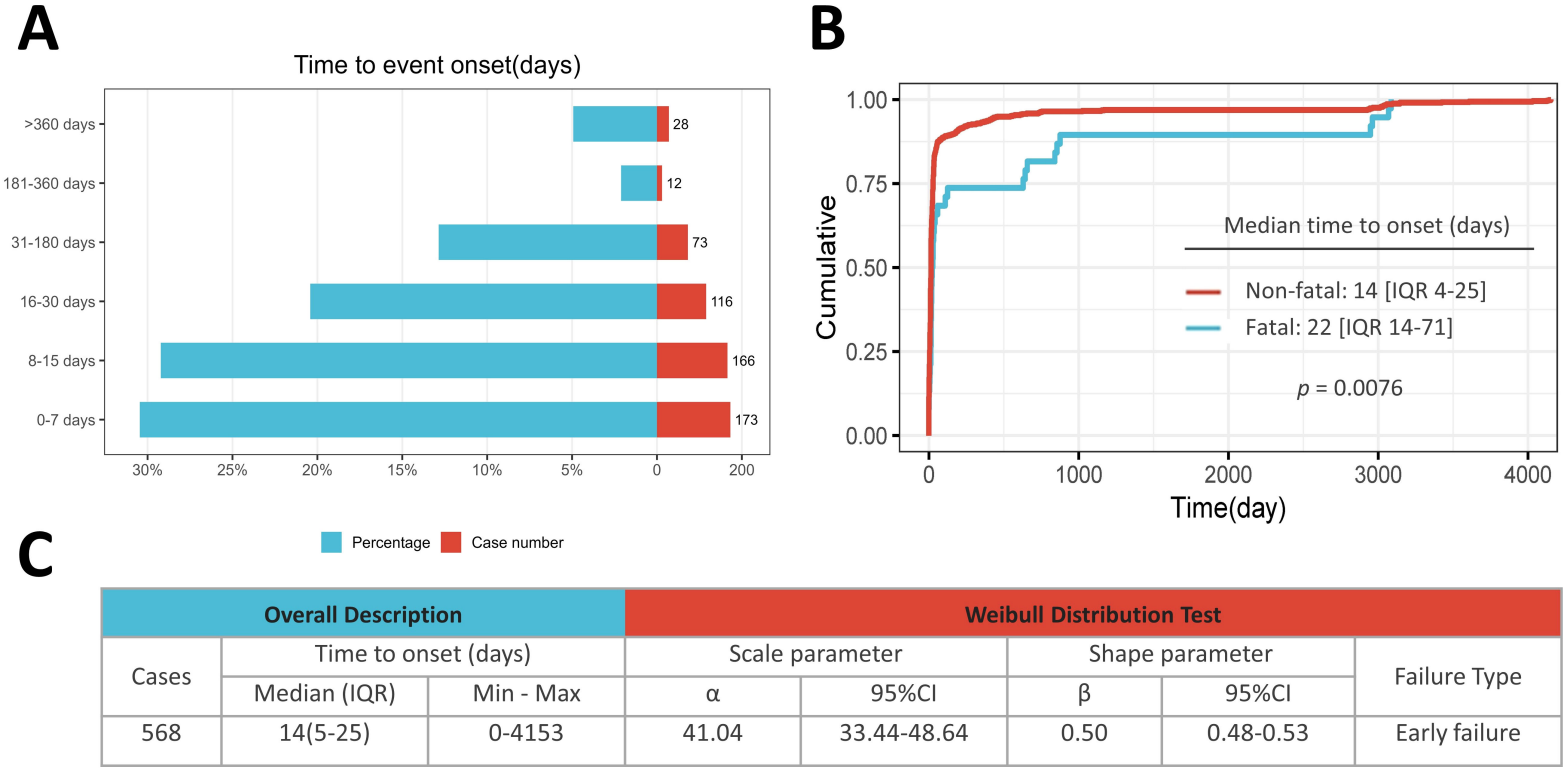
Time to onset analysis of sipuleucel-T-related cardiovascular adverse events. **(A)** Stratified distribution of time to onset for sipuleucel-T-related cardiovascular adverse events (CVAEs); **(B)** Cumulative distribution curves for time to onset of fatal versus nonfatal CVAEs associated with sipuleucel-T treatment; **(C)** Overall description of CVAE onset timing and results of Weibull distribution tests.

There were 568 cases (76.45%) of sipuleucel-T-associated CVAEs with documented onset times in the FAERS database, with a median onset time of 14 days (IQR, 5–25). Most CVAEs occurred during the first week after the initial sipuleucel-T therapy (173 cases, 30.46%), and 80.11% (455 cases) developed within the first month (**Figure 3A**). Moreover, **Figure 3B** compares the time to onset of fatal CVAE cases with that of non-fatal CVAE cases. Overall, the median time to onset of fatal CVAEs associated with sipuleucel-T was 22 days (IQR, 14–71), which was significantly later than that of non-fatal CVAE cases (14 days [IQR, 4-25]; *p* = 0.0076). It is worth noting that, as shown in **Figure 3C**, the results of the Weibull shape parameter test indicate that both the shape parameter β and the upper limit of its 95% CI are < 1. This finding suggests that sipuleucel-T-related CVAEs follow an early failure pattern, characterized by an initial increase in CVAE risk followed by a subsequent decrease over time.

## 4 Discussion

Sipuleucel-T, the first and only therapeutic cancer vaccine approved by the FDA, has represented an unprecedented advancement in the treatment paradigm for mCRPC (6, 20). However, alongside its impressive efficacy, the emergence of CVAEs associated with this novel immunotherapeutic has become a topic of considerable interest (11, 21). Although previous reports and studies have documented CVAEs related to sipuleucel-T, a significant knowledge gap remains regarding the full spectrum of potential CVAEs linked to this drug (22). The present pharmacovigilance study, which utilizes real-world data from the FAERS database, provides a comprehensive analysis of CVAEs pertinent to sipuleucel-T therapy and explores the clinical profiles as well as underlying risk factors associated with such events.

The heterogeneity between clinical trial populations and real-world patients, along with the extended durations of drug exposure in everyday practice, may limit the ability of trials to fully capture the breadth of CVAEs associated with sipuleucel-T (11, 21, 22). Results from the phase IV observational trial (PROCEED; NCT0136890) demonstrated an overall adjudicated incidence of cerebrovascular event associated with sipuleucel-T was 2.8% (22), but Dores et al. (23) argued that the PROCEED study likely underestimates true risk by failing to capture the full extent of these events. Currently, a pharmacovigilance study exploring drug-related VTE risks further confirmed that the VTE risk associated with sipuleucel-T is significantly higher than that with other agents (24). Moreover, cases of inflammatory cardiomyopathy have also been reported in the postmarketing clinical practice of sipuleucel-T (11). Overall, the spectrum of sipuleucel-T-associated CVAEs appears to be broader, and the characteristics of these events must be comprehensively considered.

Data from previous studies indicate that approximately 15.3% of mCRPC patients with sipuleucel-T-associated CVAEs experienced fatal outcomes, representing the second most common cause of death in this patient population (21). In this pharmacovigilance analysis, we identified 743 cases of sipuleucel-T-related CVAEs, with 427 cases (57.47%) classified as serious AEs, and the overall CVAE-related mortality rate was 6.3% (**Table 1**). Notably, this 6.3% mortality rate is lower than previously reported rates, a difference that likely reflects the accumulating clinical experience in managing immunotherapy-related CVAEs in recent years (25). Despite annual fluctuations in the number of reported CVAEs associated with sipuleucel-T, their proportion among all AEs remains relatively high (**Figure 1**). Although sipuleucel-T is generally considered safe and well-tolerated from a cardiovascular perspective outside of the known hypertension and cerebrovascular events reported in pivotal trials (6, 7, 9, 10), our analysis of FAERS data suggests that the incidence of serious CVAEs associated with sipuleucel-T may be underestimated compared with previous reports.

Our disproportionality analysis identified the top 3 reported CVAEs associated with sipuleucel-T therapy as hypertension (n = 107, 11.69%), blood pressure increased (n = 74, 8.09%), and deep vein thrombosis (n = 63, 6.89%) (**Supplementary Table 3**). Consistent with findings from pivotal clinical trials of sipuleucel-T (6, 22), hypertension was frequently observed. Disproportionality analysis revealed that the most significant CVAEs for sipuleucel-T at the SMQ level were VTE (n = 149, ROR_025_ = 2.89; IC_025_ = 1.51) (**Figure 2**), further supporting concerns that it may increase the risk of VTE in mCRPC patients (24).

Furthermore, specific sipuleucel-T-associated CVAEs at the PT level were first systematically disclosed in the present study, comprising a total of 27 disproportionate signals (**Supplementary Figure 1**). Beyond the already recognized cerebrovascular accidents and hypertension, sensitivity analysis identified atrial fibrillation (ROR_025_ = 1.41; IC_025_ = 0.49), cardiac failure congestive (ROR_025_ = 1.84; IC_025_ = 0.88), hypertension (ROR_025_ = 2.35; IC_025_ = 1.22), acute myocardial infarction (ROR_025_ = 1.77; IC_025_ = 0.84), transient ischemic attack (ROR_025_ = 1.78; IC_025_ = 0.85), deep vein thrombosis (ROR_025_ = 2.64; IC_025_ = 1.39), and pulmonary embolism (ROR_025_ = 1.62; IC_025_ = 0.69) as disproportionately reported in patients receiving sipuleucel-T therapy (**Table 2**). It is important to note that disproportionality analysis is a hypothesis-generating activity representing only a potential statistical association, not causation. Nevertheless, such analysis may provide clinicians with vigilant evidence for the early identification and intervention of potential CVAEs associated with sipuleucel-T therapy. Moreover, patients with mCRPC are typically older and often have coexisting hypertension and diabetes. These comorbidities contribute directly to the risk of cerebrovascular events by promoting atherosclerotic progression and disrupting normal coagulation (26). Additionally, prostate cancer itself, androgen-deprivation therapy (and its duration), polypharmacy, and tumor burden are also well-established risk factors for CVAEs in this population (27–30). Therefore, results should be interpreted with caution, and establishing definitive causation requires further research and validation.

As shown in **Table 4**, our logistic regression analysis revealed that patients aged 75 years or older had a significantly higher proportion of reported CVAEs (OR = 1.61, 95%CI: 1.46-1.79), emphasizing the need for periodical cardiac and thrombotic monitoring in this age group. Moreover, among patients receiving sipuleucel-T, the concurrent use of five or more additional medications was associated with a significant increase in the proportion of reports documenting CVAEs (OR = 1.77, 95%CI: 1.49-2.13). This observation underscores the critical importance of meticulous medication management, particularly for individuals on multiple concurrent therapeutic regimens. Our study findings further support the evidence that advanced age (age ≥ 75) and polypharmacy may play a significant role in CVAEs development following sipuleucel-T administration (28, 29).

Currently, extensive clinical evidence implicates CVAEs in patients treated with sipuleucel-T, but no studies have systematically elucidated the underlying mechanisms responsible for this pathological condition. The temporal pattern of CVAEs associated with sipuleucel-T appears to shed light on its multiphase immunopathological process, which aligns with the pharmacokinetic and pharmacodynamic characteristics of sipuleucel-T. Most CVAEs occurred within the first week (173 cases, 30.5%) and followed an early-onset pattern (**Figures 3A, C**), consistent with acute systemic immune activation. Infusion of PA2024-loaded APCs rapidly engages T cells, triggering a cytokine surge (IL-6, TNF-α, IFN-γ) that begins within hours and peaks at 1–2 weeks (31, 32). This inflammatory milieu upregulates endothelial adhesion molecules, promotes leukocyte-platelet aggregation, and initiates immunothrombosis via neutrophil extracellular traps (NETs), tissue factor expression, and complement activation. This thromboinflammatory loop likely underlies early, often nonfatal, events such as transient ischemia or venous thromboembolism (33).

In contrast, fatal CVAEs exhibited a significantly delayed median onset (22 days *vs.* 14 days for nonfatal CVAEs)(**Figure 3B**), suggesting a biphasic pathological response. The initial phase (days 7–14) may reflect APC-driven innate activation and microvascular injury (31). The second, more critical phase (days 15–28) coincides with the peak expansion of antigen-specific CD4^+^ and CD8^+^ T cells, sustaining high levels of IFN-γ, IL-17, and cytotoxic mediators (11, 34). While direct histopathological evidence of myocardial T-cell infiltration following sipuleucel-T is lacking, the well-characterized mechanism of immune checkpoint inhibitor–associated myocarditis provides a cogent analogy: activated CD8^+^ T cells can infiltrate the heart and cause fulminant injury (35). By extension, prolonged T-cell–mediated endothelial damage, synergized with persistent NETosis and coagulopathy, may precipitate occlusive thrombosis in critical vascular beds, culminating in the observed peak of fatal events around week 3.

Notably, a minority of CVAEs (7%) occurred beyond 180 days. Although speculative, this late risk could be rooted in antigen spreading during ex vivo APC culture (36). Dendritic cells may phagocytose not only PA2024 but also co-existing self-antigens, including low-abundance cardiac proteins such as troponins. Cross-presentation of these epitopes might prime autoreactive T cells, a phenomenon documented in other vaccine contexts. Durable antibody responses to PA2024 and PAP persisting up to 26 weeks support sustained adaptive immunity (31). Thus, while direct evidence of anti-cardiac autoimmunity is absent, persistent immune activation offers a plausible framework for delayed cardiomyocyte injury or vascular inflammation in susceptible individuals.

In summary, CVAEs associated with sipuleucel-T appear to evolve through three immunologically distinct phases: (1) early thromboinflammation driven by innate activation, (2) delayed, T-cell–amplified injury potentially leading to fatal thrombosis, and (3) rare late-onset events possibly linked to epitope spreading and autoimmunity. These findings underscore that cardiologists, oncologists, and pharmacists should pay particular attention to the potential CVAEs linked to sipuleucel-T, as these events can be serious or even fatal. Cardiovascular risk monitoring should extend well beyond the immediate post-infusion period, encompassing structured monitoring in the subacute (weeks 2-4) and consideration of long-term vigilance in high-risk patients, potentially guided by symptom assessment and selective biomarker screening. Furthermore, investigation into optimized therapeutic strategies that mitigate off-target organ inflammation—while preserving the efficacy of immunotherapy—will help to further improve quality of life among patients with mCRPC (37).

The main strength of the present study is that it represents the first systematic characterization of the spectrum of sipuleucel-T-associated CVAEs, clarifying their time to onset patterns and relevant risk factors, thereby providing crucial pharmacovigilance evidence for the optimal management of mCRPC patients. Moreover, like other pharmacovigilance studies relying on passive surveillance reporting systems, the present study acknowledged certain limitations. First, the FAERS database is inherently constrained by issues of voluntary (underreporting) and duplicate reporting (despite efforts to exclude duplicate reports), reporting bias, and variable report quality (38, 39). Second, confounding factors—such as advanced age, preexisting cardiovascular conditions, prior exposure to chemotherapy, disease severity, and baseline patient characteristics—may limit the detection of CVAEs in a standard comparison. Third, disproportionality analysis does not quantify absolute risk or establish causality, it only estimates the strength of safety signals, prompting clinicians and pharmacists to remain vigilant regarding such events (40). Therefore, well-designed prospective clinical trials are still required to validate the causal association between sipuleucel-T and the CVAEs development.

## 5 Conclusions

In conclusion, based on real-world data leveraging the FAERS, this study provides an overview of the clinical spectrum, time-to-onset patterns, and risk factors of sipuleucel-T-related CVAEs, while also identifying potential CVAEs not previously recognized in clinical trials. Comprehensive analysis of the FAERS database revealed that sipuleucel-T-related CVAEs are more common than previously appreciated, with an overall frequency of 15.49%. Additionally, this study represents the first systematic identification of cardiac and/or vascular safety signals associated with sipuleucel-T at both the SMQ and PT levels. Older age (≥ 75 years) and polypharmacy (concomitant use of ≥ 5 medications) were identified as significant risk factors for sipuleucel-T-related CVAEs. Given the increasing clinical relevance of cardio-oncology, these findings highlight the need for heightened awareness and proactive management of potential CVAEs associated with sipuleucel-T, particularly in the aforementioned high-risk populations. Further prospective epidemiological studies are warranted to validate the causal relationship between sipuleucel-T and CVAEs, and experimental studies are needed to explore the underlying pathophysiological mechanisms of these events.

## Data Availability

The original contributions presented in the study are included in the article/**Supplementary Material**. Further inquiries can be directed to the corresponding authors.

## Supplementary material

Supplementary Figure 1 Disproportionality analysis of significant cardiovascular adverse events associated with sipuleucel-T at the Preferred Term (PT) level. A signal was considered significant if three criteria were met simultaneously: the lower limit of the reporting odds ratio (ROR) exceeded 1, the lower bound of the 95% confidence interval for the information component (IC) was greater than 0, and the number of cases was ≥3.

## References

[1] Siegel RL Giaquinto AN Jemal A . Cancer statistics, 2024. CA Cancer J Clin. (2024) 74:12–49. doi: 10.3322/caac.21820, PMID: 38230766

[2] Gravis G . Metastatic prostate cancer management: 20 years of progress. Lancet Oncol. (2023) 24:416–7. doi: 10.1016/S1470-2045(23)00167-5, PMID: 37142365

[3] Dwyer L Leslie C Mellor R Scheinberg T Taylor RA Horvath LG . Immunotherapy in metastatic prostate cancer. Ther Adv Med Oncol. (2025) 17:17588359251347857. doi: 10.1177/17588359251347857, PMID: 40621483 PMC12227887

[4] Kulasegaran T Oliveira N . Metastatic castration-resistant prostate cancer: advances in treatment and symptom management. Curr Treat Options Oncol. (2024) 25:914–31. doi: 10.1007/s11864-024-01215-2, PMID: 38913213 PMC11236885

[5] Garje R Riaz IB Naqvi SAA Rumble RB Taplin ME Kungel TM . Systemic therapy in patients with metastatic castration-resistant prostate cancer: ASCO guideline update. J Clin Oncol. (2025) 43:2311–34. doi: 10.1200/JCO-25-00007, PMID: 40315400

[6] Kantoff PW Higano CS Shore ND Berger ER Small EJ Penson DF . Sipuleucel-T immunotherapy for castration-resistant prostate cancer. N Engl J Med. (2010) 363:411–22. doi: 10.1056/NEJMoa1001294, PMID: 20818862

[7] Beer TM Bernstein GT Corman JM Glode LM Hall SJ Poll WL . Randomized trial of autologous cellular immunotherapy with sipuleucel-T in androgen-dependent prostate cancer. Clin Cancer Res. (2011) 17:4558–67. doi: 10.1158/1078-0432.CCR-10-3223, PMID: 21558406

[8] Pieczonka CM Telonis D Mouraviev V Albala D . Sipuleucel-T for the treatment of patients with metastatic castrate-resistant prostate cancer: considerations for clinical practice. Rev Urol. (2015) 17:203–10., PMID: 26839517 PMC4735666

[9] Small EJ Schellhammer PF Higano CS Redfern CH Nemunaitis JJ Valone FH . Placebo-controlled phase III trial of immunologic therapy with sipuleucel-T (APC8015) in patients with metastatic, asymptomatic hormone refractory prostate cancer. J Clin Oncol. (2006) 24:3089–94. doi: 10.1200/JCO.2005.04.5252, PMID: 16809734

[10] Higano CS Schellhammer PF Small EJ Burch PA Nemunaitis J Yuh L . Integrated data from 2 randomized, double-blind, placebo-controlled, phase 3 trials of active cellular immunotherapy with sipuleucel-T in advanced prostate cancer. Cancer. (2009) 115:3670–9. doi: 10.1002/cncr.24429, PMID: 19536890

[11] Moey MYY Jiwani RA Takeda K Prenshaw K Kreeger RW Inzerillo J . Sipuleucel-T associated inflammatory cardiomyopathy: a case report and observations from a large pharmacovigilance database. ESC Heart Fail. (2021) 8:3360–8. doi: 10.1002/ehf2.13400, PMID: 33938158 PMC8318408

[12] Liu W Gao F Song X Chen H She Y Liu J . Emerging cardiovascular toxicity associated with CDK4/6 inhibitors: real-world insights from the FDA adverse event reporting system. Front Pharmacol. (2025) 16:1558128. doi: 10.3389/fphar.2025.1558128, PMID: 40520154 PMC12162638

[13] Fusaroli M Salvo F Begaud B AlShammari TM Bate A Battini V . The reporting of a disproportionality analysis for drug safety signal detection using individual case safety reports in pharmacoVigilance (READUS-PV): development and statement. Drug Saf. (2024) 47:575–84. doi: 10.1007/s40264-024-01421-9, PMID: 38713346 PMC11116242

[14] Fusaroli M Salvo F Begaud B AlShammari TM Bate A Battini V . The REporting of A disproportionality analysis for drUg safety signal detection using individual case safety reports in pharmacoVigilance (READUS-PV): explanation and elaboration. Drug Saf. (2024) 47:585–99. doi: 10.1007/s40264-024-01423-7, PMID: 38713347 PMC11116264

[15] Agha RA Mathew G Rashid R Kerwan A Al-Jabir A Sohrabi C . Transparency In The reporting of Artificial INtelligence – the TITAN guideline. Premier J Sci. (2025) 10:100082. doi: 10.70389/PJS.100082

[16] Muganurmath CS Curry AL Schindzielorz AH . Causality assessment of olfactory and gustatory dysfunction associated with intranasal fluticasone propionate: application of the bradford hill criteria. Adv Ther. (2018) 35:173–90. doi: 10.1007/s12325-018-0665-5, PMID: 29396682 PMC5818548

[17] Xia S Gong H Zhao Y Guo L Wang Y Ma R . Tumor lysis syndrome associated with monoclonal antibodies in patients with multiple myeloma: A pharmacovigilance study based on the FAERS database. Clin Pharmacol Ther. (2023) 114:211–9. doi: 10.1002/cpt.2920, PMID: 37086211

[18] Liu W Ye X Shan H Wang M Wang Y Guo Z . Unraveling the spectrum of ocular toxicity with oxaliplatin: clinical feature analysis of cases and pharmacovigilance assessment of the US food and drug administration adverse event reporting system database. Clin Ther. (2024) 46:1049–58. doi: 10.1016/j.clinthera.2024.09.019, PMID: 39428274

[19] Liu W Song X Du Q Liu J . Emerging causes of anticancer therapies-induced Stevens-Johnson syndrome and toxic epidermal necrolysis: evidence from disproportionality analysis of the FDA adverse event reporting system. Front Immunol. (2025) 16:1646038. doi: 10.3389/fimmu.2025.1646038, PMID: 40936888 PMC12420621

[20] Zhou W Lu X Tian F Luo Q Zhou W Yang S . Vaccine therapies for prostate cancer: current status and future outlook. Vaccines (Basel). (2024) 12:1384. doi: 10.3390/vaccines12121384, PMID: 39772046 PMC11679746

[21] Dores GM Bryant-Genevier M Perez-Vilar S . Adverse events associated with the use of sipuleucel-T reported to the US food and drug administration’s adverse event reporting system, 2010-2017. JAMA Netw Open. (2019) 2:e199249. doi: 10.1001/jamanetworkopen.2019.9249, PMID: 31411714 PMC6694390

[22] Higano CS Armstrong AJ Sartor AO Vogelzang NJ Kantoff PW McLeod DG . Real-world outcomes of sipuleucel-T treatment in PROCEED, a prospective registry of men with metastatic castration-resistant prostate cancer. Cancer. (2019) 125:4172–80. doi: 10.1002/cncr.32445, PMID: 31483485 PMC6856402

[23] Dores GM Niu MT Izurieta HS . Potential underestimation of cerebrovascular events in the PROVENGE Registry for the Observation, Collection, and Evaluation of Experience Data. Cancer. (2020) 126:2934–5. doi: 10.1002/cncr.32786, PMID: 32154909

[24] Cai X Chen G Wang H Wang L Hu C . Pharmacovigilance insights into drug-associated venous thromboembolism. Int J Surg. (2025) 111:7677–85. doi: 10.1097/JS9.0000000000002931, PMID: 40607923 PMC12626569

[25] Patel M Hudson O Han J Kondapalli L Arora G Hawi R . Update on immunotherapy cardiotoxicity: checkpoint inhibitors, CAR T, and beyond. Curr Treat Options Oncol. (2023) 24:1489–503. doi: 10.1007/s11864-023-01130-y, PMID: 37624557

[26] Liu Y Li J Dou Y Ma H . Impacts of type 2 diabetes mellitus and hypertension on the incidence of cardiovascular diseases and stroke in China real-world setting: a retrospective cohort study. BMJ Open. (2021) 11:e053698. doi: 10.1136/bmjopen-2021-053698, PMID: 34845072 PMC8634005

[27] Mahajan A Brunson A Adesina O Keegan THM Wun T . The incidence of cancer-associated thrombosis is increasing over time. Blood Adv. (2022) 6:307–20. doi: 10.1182/bloodadvances.2021005590, PMID: 34649273 PMC8753193

[28] Cebe T Kızılyel F . Risk of senescence, polypharmacy, and their outcomes in elderly cardiovascular disease patients. Adv Pharmacol. (2025) 104:351–92. doi: 10.1016/bs.apha.2025.02.011, PMID: 40716935

[29] Klil-Drori AJ Yin H Tagalakis V Aprikian A Azoulay L . Androgen deprivation therapy for prostate cancer and the risk of venous thromboembolism. Eur Urol. (2016) 70:56–61. doi: 10.1016/j.eururo.2015.06.022, PMID: 26138040

[30] Pinthus JH Duivenvoorden WCM . Cardiovascular risk in ADT recipients: does the type of ADT matter? Prostate Cancer Prostatic Dis. (2024) 27:435–7. doi: 10.1038/s41391-024-00832-0, PMID: 38664503

[31] Sheikh NA Petrylak D Kantoff PW Dela Rosa C Stewart FP Kuan LY . Sipuleucel-T immune parameters correlate with survival: an analysis of the randomized phase 3 clinical trials in men with castration-resistant prostate cancer. Cancer Immunol Immunother. (2013) 62:137–47. doi: 10.1007/s00262-012-1317-2, PMID: 22865266 PMC3541926

[32] Strauss J Madan RA Figg WD . Evaluating immune responses after sipuleucel-T therapy. Cancer Biol Ther. (2015) 16:1119–21. doi: 10.1080/15384047.2015.1056417, PMID: 26054644 PMC4622939

[33] Wang Y Mulder IA Westendorp WF Coutinho JM van de Beek D . Immunothrombosis in acute ischemic stroke. Stroke. (2025) 56:553–63. doi: 10.1161/STROKEAHA.124.048137, PMID: 39479751

[34] Laus R Yang DM Ruegg CL Shapero MH Slagle PH Small EJ . Dendritic cell immunotherapy of prostate cancer: preclinical models and early clinical experience. Cancer Res Ther Control. (2001) 11:1–10.

[35] Moslehi J Lichtman AH Sharpe AH Galluzzi L Kitsis RN . Immune checkpoint inhibitor-associated myocarditis: manifestations and mechanisms. J Clin Invest. (2021) 131:e145186. doi: 10.1172/JCI145186, PMID: 33645548 PMC7919710

[36] Sidaway P . Immunotherapy: Sipuleucel-T induces humoral antigen spread in patients with mCRPC. Nat Rev Urol. (2015) 12:181. doi: 10.1038/nrurol.2015.33, PMID: 25708576

[37] Moningi S Nguyen PL . Uncontrolled cardiovascular risk factors in prostate cancer patients: are we leaving too much on the table? JACC CardioOncol. (2023) 5:82–4. doi: 10.1016/j.jaccao.2023.01.001, PMID: 36875903 PMC9982199

[38] Alvarez-Requejo A Carvajal A Bégaud B Moride Y Vega T Arias LH . Under-reporting of adverse drug reactions. Estimate based on a spontaneous reporting scheme and a sentinel system. Eur J Clin Pharmacol. (1998) 54:483–8. doi: 10.1007/s002280050498, PMID: 9776440

[39] Yan X Li Z Jiang A Chen J Huang X Hajdu A . Immunotherapy-induced cholestasis in cancer: insights from the two real-world pharmacovigilance databases of FAERS and VigiBase. Int J Surg. (2025) 111:5105–21. doi: 10.1097/JS9.0000000000002607, PMID: 40479008

[40] Almenoff JS Pattishall EN Gibbs TG DuMouchel W Evans SJ Yuen N . Novel statistical tools for monitoring the safety of marketed drugs. Clin Pharmacol Ther. (2007) 82:157–66. doi: 10.1038/sj.clpt.6100258, PMID: 17538548

