# Supplementary figures and images for: Unveiling the hidden cardiovascular risk of sipuleucel-T: a pharmacovigilance analysis using the FDA Adverse Event Reporting System, 2010–2025
Source: Front Immunol. 2026 Jan 20;16:1716090. doi: 10.3389/fimmu.2025.1716090 (PMC12864385; doi:10.3389/fimmu.2025.1716090)

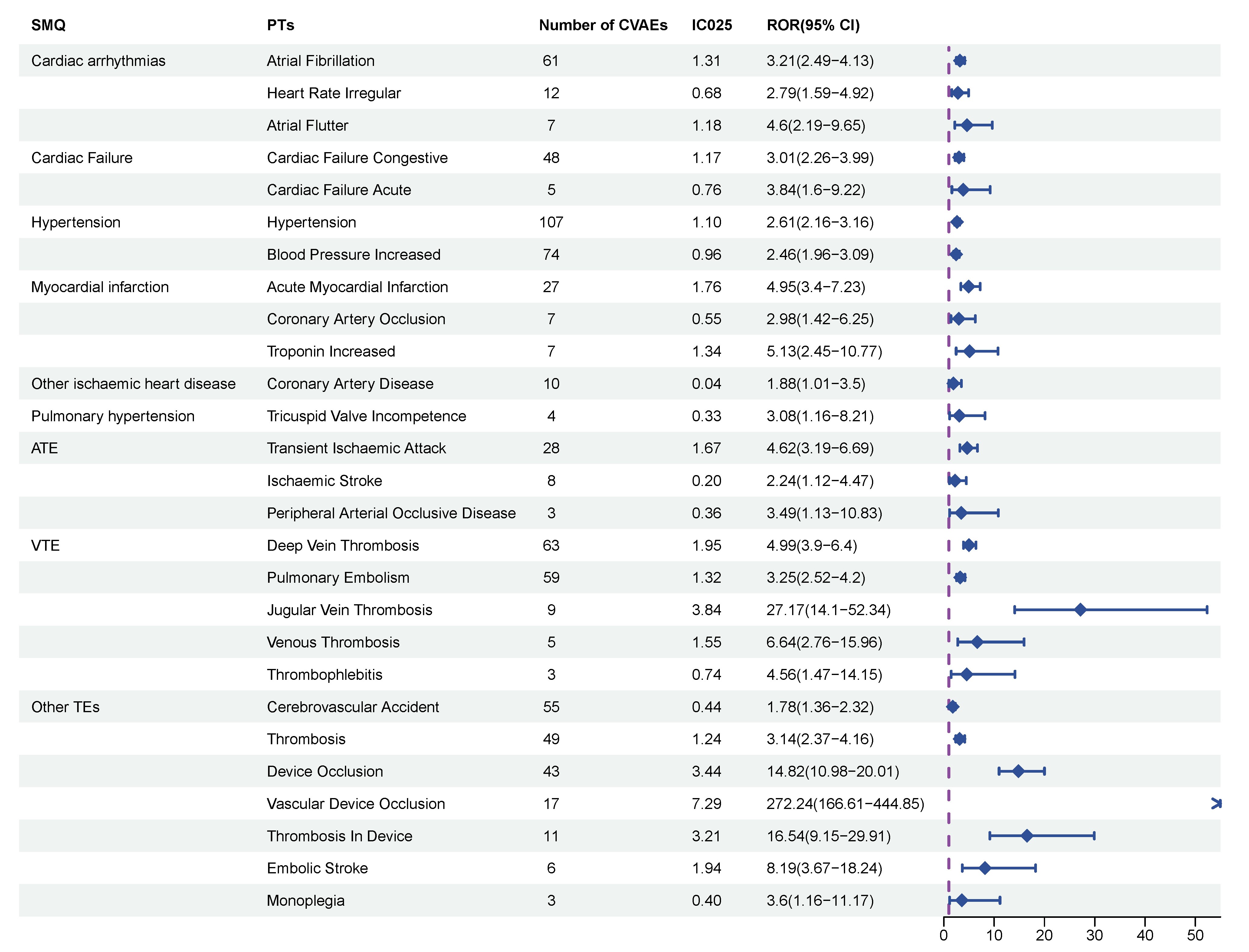

Supplement: Supplementary Figure 1 — Disproportionality analysis of significant cardiovascular adverse events associated with sipuleucel-T at the Preferred Term (PT) level. A signal was considered significant if three criteria were met simultaneously: the lower limit of the reporting odds ratio (ROR) exceeded 1, the lower bound of the 95% confidence interval for the information component (IC) was greater than 0, and the number of cases was ≥3. [file Image1.jpeg]
