# Supplementary material for: Unveiling the hidden cardiovascular risk of sipuleucel-T: a pharmacovigilance analysis using the FDA Adverse Event Reporting System, 2010–2025
Source: Front Immunol. 2026 Jan 20;16:1716090. doi: 10.3389/fimmu.2025.1716090 (PMC12864385; doi:10.3389/fimmu.2025.1716090)
Supplement: Supplementary file 3 [file Table2.docx]

| **Algorithms** | **Calculation formulas** | **Criteria** |
| --- | --- | --- |
| ROR | $ROR=\frac{(a/c)}{(b/d)}=\frac{ad}{bc}$ | 95%CI>1，a≥3 |
|  | $95\%CI=e^{ln(ROR)\pm1.96\sqrt{(\frac{1}{a}+\frac{1}{b}+\frac{1}{c}+\frac{1}{d})}}$ |  |
| BCPNN | IC =${log}_{2}\frac{a(a+b+c+d)}{(a+b)(a+c)}$ | IC_025_>0 |
|  | E(IC)=${log}_{2}\frac{(a+\gamma11)(a+b+c+d+\alpha)(a+b+c+d+\beta)}{（a+b+c+d+\gamma）(a+b+\alpha1)(a+c+\beta1)}$ |  |
|  | V(IC) =$\frac{1}{{(ln2)}^{2}}\{\left[ \frac{\left( a+b+c+d \right)-a+\gamma-\gamma11}{\left( a+\gamma11 \right)\left( 1+a+b+c+d+\gamma\right)} \right]+\left[ \frac{\left( a+b+c+d \right)-\left( a+b \right)+\alpha-\alpha1}{\left( a+b+\alpha1 \right)\left( 1+a+b+c+d+\alpha\right)} \right]+\left[ \frac{\left( a+b+c+d \right)-\left( a+c \right)+\beta-\beta1}{\left( a+c+\beta1 \right)\left( 1+a+b+c+d+\beta\right)} \right]\}$ |  |
|  | $\gamma=\gamma11\frac{(a+b+c+d+\alpha)(a+b+c+d+\beta)}{(a+b+\alpha1)(a+c+\beta1)}$ |  |
|  | IC_025_*=E(IC)-2*$\sqrt{V(IC)}$ |  |

**Supplementary Table 2.** The major algorithms used for signal detection.

**Abbreviation:** ROR, reporting odds ratio; BCPNN, Bayesian confidence propagation neural network; CI, confidence interval; IC, information component; IC_025_, the lower limit of the 95% one-sided CI of the IC;

**Equation:** *a*, number of cardiovascular adverse events of sipuleucel-T alone; *b*, number of other adverse events of sipuleucel-T alone; *c*, number of cardiovascular adverse events of other drugs except for sipuleucel-T; *d*, number of other adverse events of other drugs except for sipuleucel-T; α1=β1=1；α=β=2；γ11=1.
