# Supplementary material for: Unveiling the hidden cardiovascular risk of sipuleucel-T: a pharmacovigilance analysis using the FDA Adverse Event Reporting System, 2010–2025
Source: Front Immunol. 2026 Jan 20;16:1716090. doi: 10.3389/fimmu.2025.1716090 (PMC12864385; doi:10.3389/fimmu.2025.1716090)
Supplement: Supplementary file 5 [file Table4.docx]

**Supplementary Table 4**. Top ten concomitant medications with sipuleucel-T in cases of cardiovascular adverse events.

| **Concomitant drugs** | **N (%)** | **CVAEs (FDA Label information)** |
| --- | --- | --- |
| Leuprolide | 106 (14.27%) | Increased risk of developing myocardial infarction, sudden cardiac death and stroke has been reported in association with use of GnRH agonists in men. The risk appears low based on the reported odds ratios, and should be evaluated carefully along with cardiovascular risk factors when determining a treatment for patients with prostate cancer. Patients receiving a GnRH agonist should be monitored for symptoms and signs suggestive of development of cardiovascular disease and be managed according to current clinical practice. |
| Aspirin | 92 (12.38%) | Not common |
| Metoprolol | 87 (11.71%) | - Common: Cold extremities, bradycardia, palpitations; - Occasionally: Transient exacerbation of heart failure, cardiogenic shock in patients with acute myocardial infarction; - Rare symptoms: Prolonged atrioventricular conduction time, arrhythmia, syncope; - Unknown: Gangrene in patients with severe peripheral vascular disease. |
| Lisinopril | 69 (9.29%) | Not common, lisinopril can cause symptomatic hypotension, sometimes complicated by oliguria, progressive azotemia, acute renal failure or death. |
| Atorvastatin | 63 (8.48%) | Not common. For patients who have experienced recent strokes and transient ischemic attacks, the risk of hemorrhagic stroke should be vigilantly monitored. |
| Tamsulosin | 57 (7.67%) | Not common |
| Prednisone | 53 (7.13%) | Not common |
| Amlodipine | 49 (6.59%) | - Hypotension. Symptomatic hypotension is possible, particularly in patients with severe aortic stenosis. - Increased Angina or Myocardial Infarction. Worsening angina and acute myocardial infarction can develop after starting or increasing the dose of amlodipine, particularly in patients with severe obstructive coronary artery disease. |
| Colecalciferol/Vitamin D3 | 43 (5.79%) | Hypertension, arrhythmia |
| Acetaminophen | 40 (5.38%) | Hypertension, hypotension |

Note: CVAEs: cardiovascular adverse events; GnRH, gonadotropin-releasing hormone.
